# A Treatise on Dislocation of the Shoulder

**Published:** 1850-03

**Authors:** 


*A Treatise on Dislocation of the Shoulder.* By George O. Jarvis, M. D., Author of Lectures on Fractures and Dislocations, &c., &c.; *together with important cases illustrating the benefits of the Adjuster.* Compiled by George Kellogg, A. M.

This is the title of a well-written pamphlet of about thirty pages, detailing a single case of dislocation of the shoulder, with the head of the humerus in the axilla, together with a mention of other cases of injury to the bones and joints. It comes to us as a recommendation of the instrument well known as "Jarvis' Surgical Adjuster," which was the apparatus relied upon and used with success in the instances of which an account is given by our author. It is presented, as stated in the preface, "at the solicitation of many members of the profession," and is thought by the compiler, Mr. George Kellogg, to demonstrate "the indispensable necessity of the adjuster," while he states his disinterested motive in making the publication, to be, a desire "to promote the usefulness and unfailing success of the whole body of practicing surgeons."

If the instrument thus strenuously advocated possess the merits ascribed to it by its manufacturer, a *desideratum* is doubtless attained in the healing art, and on its adoption in practice the surgeon must soon banish from our midst the many cases of permanent deformity from severe injuries of the bones and joints which have heretofore been his *opprobria,* and put an end to costly and vexatious suits for malpractice, by which he is too frequently annoyed at the present day.

The author, Dr. Jarvis, prior to entering on an account of the case of dislocation of the shoulder, on which the essay is based, undertakes to lay down the principles on which all such injuries should be treated, and claims for the adjuster the ability to carry out the different indications, without regard to the character, extent, or duration of such injuries. The Dr. very truly states, that there is a difference between the pathology of a recent and old luxation of the shoulder joint, but still insists that the same instrument is equal alike to the reduction, unaided by the adjuvants usually resorted to, such as venesection, tartar emetic, the warm bath, and other means for the production of muscular relaxation, which have been commonly considered necessary in cases at all difficult of reduction. On the contrary, he seems to insist that much strength of the muscles is necessary (and the more the better) to accomplish the object, as upon their contraction, after having been put sufficiently on the stretch to dislodge the head of the bone from its unnatural position, does the success of the treatment depend. By the instrument, extension is made in one way only, and the surgeon is deprived of the ability to change its direction when desirable, as is the case when the pullies are used, or when the heel or knee is placed in the axilla. He also discards one of the old and fundamental directions and modes of practice, that the extension should be made in the line of the dislocation, and is entirely content with bringing the head of the bone on a level with the glenoid cavity, when all extension is to be suddenly removed, and the head of the bone driven forcibly by the contraction of the muscles, through the capsular ligament, (in which the laceration is presumed to have been closed,) into its natural position.

It is presumed that the laceration in the ligaments, through which the head of the bone has escaped, is often partially closed after old luxations; and it is known that all the muscles about the shoulder, *and not alone the deltoid and supra spinatus*, have become to a great extent paralyzed, certainly *too* much so to admit of such powerful contractions as to inflict on the closed capsule "*the impetus of a blow*, from the head of the bone being forcibly driven against it," which will lacerate it " on the same principle that an arrow is driven into a board." The case related as having been operated on successfully at the Marine Hospital in Mobile, was certainly a fair one for the trial of the adjuster, as the dislocation of the shoulder was of fifty-two days' standing, and its reduction had been well attempted by other means. Still we are unwilling to throw aside appliances which have been found heretofore generally equal to the end, for one, which can only be applied in one way, and which for its success entirely depends on the strong contraction of muscles, the functions of which must be too much interfered with, to exercise so great power after having been for so long a time totally inactive.

The author gives a series of reasons why the adjuster should be used to the exclusion of other means of extension and counter-extension, which are ingenious and applicable to his invention, though he certainly loses sight of the injury which may be done to the axillary artery and veins by the powerful and long-continued force which must be applied to old and obstinate cases, no matter what apparatus is used for the purpose. He does not allude to the dislocation of the humerus with the head of the bone beneath the pectoralis muscle and below the clavicle, though we presume that he considers the adjuster equally applicable to the reduction of this as to the other varieties of luxation of the same bone.

The cases appended to the article on dislocation of the shoulder in which the adjuster was successfully applied, are three of similar injury, one of which was of four years' standing, one of thirty-nine days, with fracture of the upper part of the humerus, a case of oblique fracture of the tibia and fibula, one of compound luxation of the thumb, one of luxation of the metatarsus upon the tarsus, one of luxation of the hip, and one of united fracture of the femur with great deformity. In the latter case the bone was re-fractured by the adjuster, "and the limb dressed on the double inclined plane in the manner directed in Jarvis' Lectures, pp. 47, 48 and 49, for oblique fracture of the thigh," and the recovery took place "with a limb in every respect as good as the other." These cases, if they do not prove too much, are more than enough to establish the value of the instrument, which is so modified as to be applied to the injuries of all the limbs and many joints of the body.

The essay, as we have before stated, is well written, and for it we are thankful to its author and compiler; but we would be pleased to receive accounts of cases treated by the adjuster from professional men uninterested in its manufacture and sale, so that we may not by possibility regard it in the same light as Peter Pindar's razors, "*made to sell.*"

